# Benign Acute Childhood Myositis: A Three-Year Experience From a Pediatric Department in Poland

**DOI:** 10.7759/cureus.93822

**Published:** 2025-10-04

**Authors:** Bartosz Szarawarski, Mateusz Nowak, Joanna Kula-Gradzik

**Affiliations:** 1 Pediatrics, Specialist Hospital No. 2, Bytom, POL

**Keywords:** creatine kinase, gait disturbance, influenza, myalgia, myositis, viral infection

## Abstract

Benign acute childhood myositis (BACM) is a condition characterized by the sudden onset of bilateral lower limb muscle pain and gait disturbances, usually appearing a few days after a viral flu-like illness. Although most often linked to influenza virus infection, various other pathogens have also been reported. The disease mainly affects children during late autumn, winter, and early spring. Laboratory findings often show temporary elevation of muscle enzymes, particularly creatine kinase (CK) and aspartate aminotransferase (AST). Despite its striking presentation, BACM is self-limiting with an excellent prognosis and typically requires only supportive care, including rest, hydration, and analgesia. A key clinical challenge is distinguishing BACM from rhabdomyolysis, a more serious condition involving extensive muscle breakdown, which can lead to worsening general condition, altered consciousness, and acute kidney injury. This retrospective study analyzes the clinical and laboratory features of children diagnosed with BACM and hospitalized between April 2022 and April 2025 in the Clinical Department of Pediatrics in Bytom, Poland. To identify similarities and differences, the findings were compared with data from other studies in international medical databases. Overall, the study conclusions were consistent with previously published literature.

## Introduction

Myositis is an inflammatory process that leads to the destruction of muscle tissue. Clinically, it presents with pain, tenderness, and impaired function of the affected muscles. It is a heterogeneous condition with multiple possible causes, including infections, genetic syndromes, autoimmune diseases, congenital metabolic disorders, neuropathies, muscular dystrophies, toxins, neoplasms, endocrine dysfunctions, and electrolyte imbalances. In many cases, the underlying cause remains idiopathic, often involving individuals with a genetic predisposition. One clinical variant encountered by pediatricians is benign acute childhood myositis (BACM). Although BACM generally requires only supportive care, careful monitoring of the patient’s condition and clinical course is important. In atypical cases, a broader differential diagnosis is needed, with particular attention to ruling out rhabdomyolysis, a potentially life-threatening disorder that can present with similar but more severe symptoms.

Benign acute childhood myositis

Clinical Manifestations

BACM usually presents with the sudden onset of bilateral, symmetrical, and severe pain in the calves (gastrocnemius and soleus muscles), accompanied by gait disturbances ranging from tiptoe walking to complete refusal to bear weight [1-3]. These symptoms are typically preceded by a flu-like illness with fever, malaise, nasal congestion, cough, and sometimes gastrointestinal complaints. Muscular symptoms generally appear two to five days after the onset of infection, often during the recovery phase [2,4,5]. The pathogenesis of muscle injury and necrosis is thought to involve direct viral invasion of muscle fibers, although immune-mediated mechanisms have also been suggested [6-8]. Electromyography (EMG) is rarely performed during BACM episodes, and findings are usually normal or show only minimal myopathic changes. Muscle biopsy, when performed, may reveal focal necrosis with interstitial inflammation [3,5]. Calf tenderness and pain worsen with movement, particularly during ankle dorsiflexion [9,10]. Symptoms are often more noticeable after rest, such as in the morning after sleep [8]. Except for mild swelling of the affected muscles, other signs of local inflammation, such as warmth or redness, are absent [5,10,11]. Although pain may occasionally extend to the anterior thighs or upper limbs, the calves remain the most commonly affected site [12]. Gait abnormalities include toe-walking, a wide-based stiff-legged gait, dragging of the feet, and, in some cases, complete refusal to walk [9-13]. Neurological examination is normal, with preserved strength, tone, reflexes, and joint mobility [1,4,8,13]. Apparent weakness or plantar flexion of the feet is attributable to pain and protective posturing, rather than true motor deficit [1,8,10,11].

Epidemiology

BACM mainly affects preschool- and early school-aged children, although cases in adolescents and adults have also been reported [10,13-16]. A review published in 2024 reported a mean age of approximately 6.8 years [17]. The predominance in children may be explained by the higher susceptibility of immature muscle cells to direct viral injury [2]. There is a clear male predominance, with male-to-female ratios ranging from 2:1 to 6:1 [17,18]. Proposed explanations include higher physical activity levels in boys, genetic predisposition, or as-yet unidentified metabolic factors [2,14,15,17,19]. The incidence of BACM peaks during viral seasons, from late autumn to early spring, with seasonal rates of about 2.6 per 100,000 children (especially during epidemics) compared to 0.3 per 100,000 in other periods [2,12]. The most commonly implicated pathogen is the influenza virus, particularly type B. Other associated viruses include parainfluenza, adenovirus, rotavirus, respiratory syncytial virus (RSV), Epstein-Barr virus (EBV), cytomegalovirus (CMV), herpes simplex virus (HSV), coxsackievirus, and dengue. In rare cases, bacterial or protozoal infections, such as *Mycoplasma pneumoniae*, *Salmonella*, *Streptococcus pyogenes*, or *Toxoplasma gondii*, have been reported as causative agents [3,5,8,13-15,20].

Laboratory Investigations

Typical laboratory findings in BACM include elevated serum creatine kinase (CK), which is the most consistent abnormality, along with increased aspartate aminotransferase (AST) and, less commonly, alanine aminotransferase (ALT) and lactate dehydrogenase (LDH). Complete blood count (CBC) may reveal leukopenia, neutropenia, or thrombocytopenia, reflecting a recent viral infection [5,6,11-14]. The rise in serum CK activity is due to the release of the enzyme from damaged muscle cells during the inflammatory process. This finding helps differentiate myositis from myalgia, the latter being a prodromal symptom of influenza-like illness in which significant CK elevation is usually absent [13,14]. C-reactive protein (CRP) may be mildly elevated because of residual viral inflammation, but does not indicate direct muscle involvement [20]. Other laboratory parameters, including electrolytes, creatinine, and uric acid, are typically within normal ranges [1]. Etiologic testing may involve antigen detection, polymerase chain reaction (PCR), or serology, though identifying the specific pathogen rarely alters management.

Histopathological examination of muscle biopsy, EMG, and magnetic resonance imaging (MRI) are rarely performed because they may cause unnecessary anxiety and trauma for the child, particularly given the nonspecific clinical presentation [2,9-11]. These procedures should be reserved for atypical cases that do not follow the usual clinical pattern, where a broader differential diagnosis is required.

Clinical Course and Treatment

The cornerstone of BACM management is rest and avoidance of physical exertion during the acute phase. Bed rest is recommended, with gentle mobilization started within 24-48 hours after symptom onset [1,8,13]. Adequate hydration is essential, particularly for kidney protection in cases at risk of rhabdomyolysis. Children should be encouraged to maintain oral fluid intake, and if this is not possible, intravenous hydration should be provided [8,18,20]. Analgesics such as paracetamol, ibuprofen, or naproxen may be used to relieve significant pain [2,14,20]. Antiviral therapy directed against influenza has limited benefit, as BACM typically develops during the later phase of infection [2,6,13]. Hematologic and biochemical abnormalities are transient [1]. The condition is self-limiting with an excellent prognosis. Most patients show marked improvement within 24 hours, with full recovery usually achieved within one week, often by days 3-4 [7,21,22]. CK levels return to normal within 1-2 weeks [12,23]. Recurrence occurs in about 3%-10% of cases, often triggered by a different viral pathogen. In patients with multiple recurrences, underlying metabolic myopathies should be considered [7,22].

Rhabdomyolysis vs. BACM

The most serious complication of BACM is rhabdomyolysis, a condition characterized by massive skeletal muscle breakdown with the release of intracellular contents (CK, AST, ALT, electrolytes, myoglobin) into the bloodstream, potentially leading to multiorgan damage, particularly acute kidney injury (AKI). Although uncommon, occurring in about 3% of BACM cases, reported rates were as high as 6.8%. Unlike the focal necrosis seen in BACM, rhabdomyolysis involves more generalized and severe muscle necrosis [10]. Clinically, it presents with worsening general condition, severe pain and stiffness, painful cramps, muscle swelling, and functional impairment, most often affecting the arms, thighs, and lower back, and less commonly the calves. Additional symptoms may include fever, vomiting, and altered mental status [2,8,10]. A characteristic finding is dark, brown-colored urine (resembling black tea), caused by myoglobinuria [6,13]. This can be indirectly suggested by a positive dipstick test for hemoglobin in the urine, but confirmation requires the absence of red blood cells in the urinary sediment [6]. Rhabdomyolysis can be life-threatening if complicated by AKI. However, the overall prognosis is generally favorable [13]. There is no universally accepted serum CK threshold for diagnosing rhabdomyolysis, but the risk of complications increases when CK levels exceed 15,000-20,000 U/L. Importantly, in the presence of coexisting factors such as sepsis, dehydration, or metabolic dysfunction, AKI may develop even at much lower CK levels.

*Objectives of the Study* 

Based on available scientific sources, we conducted a study to identify specific variables in patients’ clinical presentation and compared our findings with published data to highlight similarities and differences.

## Materials and methods

In July 2025, a retrospective review was conducted of patients hospitalized in the Clinical Department of Pediatrics in Bytom between April 2022 and April 2025 who were diagnosed with infective myositis (ICD-10: M60.0). Patients with M60.8 or M60.9 diagnoses were excluded, as their disease course was atypical, as well as those with comorbidities that could confound assessment of disease progression. Only inpatients were included; patients treated exclusively in the emergency department were excluded. Diagnosis was based on clinical history, physical examination, and laboratory findings. Data were obtained through a complete documentation review, as no standardized scheme for systematic data collection was in place during admission or hospitalization. Variables collected included age, sex, duration of hospitalization, preceding symptoms, onset interval, etiologic agent, CK and AST levels (initial and peak), presence of leukopenia, neutropenia, myoglobinuria, symptom resolution time, and treatment. Reference values were as follows: WBC 4-10 × 10³/µL, neutrophils 1.5-6 × 10³/µL, platelets (PLT) 150-450 × 10³/µL, CK <190 U/L, and AST 40 U/L. Data were extracted from the hospital’s Asseco Medical Management Solutions system and analyzed using Microsoft Excel (Microsoft Corp., Redmond, WA, USA). Descriptive statistics calculated included total, maximum, minimum, mean, and median values.

## Results

**Figure 1:**
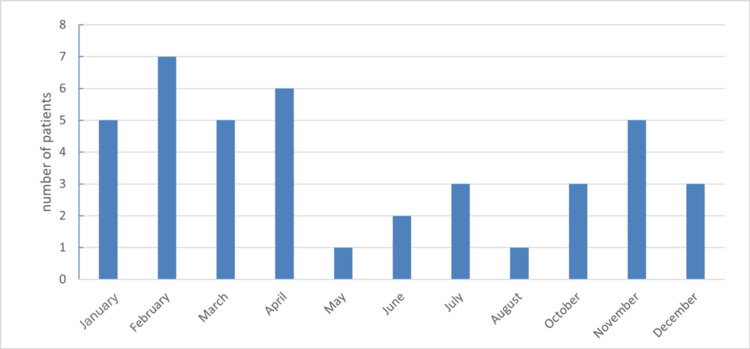
Monthly hospitalization frequency

Over the 36-month study period, 41 patients accounted for 43 hospitalizations, with two patients admitted twice in consecutive seasons. Most admissions occurred during the colder months, from November to April (72%, n = 31). The monthly distribution of hospitalizations is shown in Figure 1.

**Figure 2:**
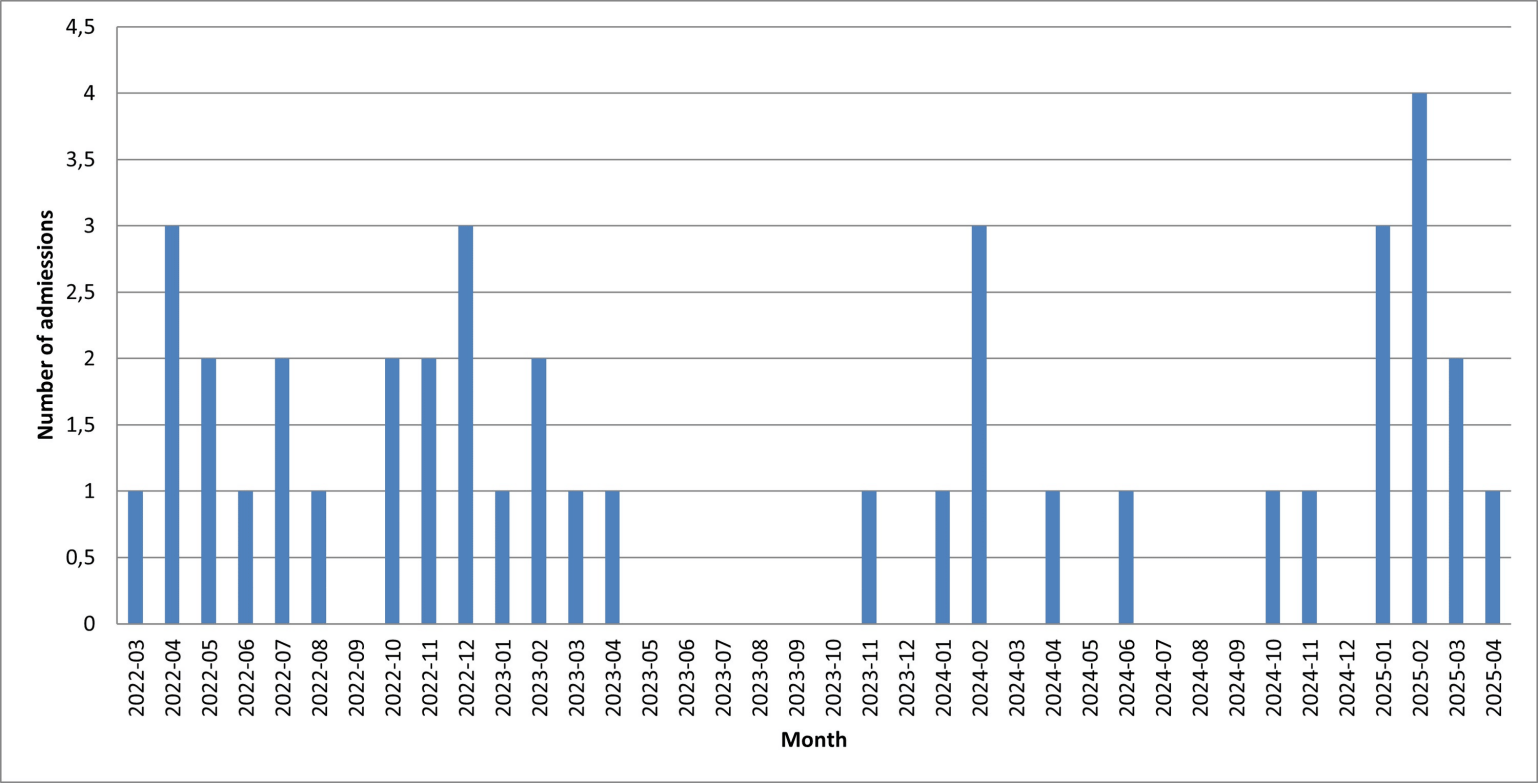
Number of hospitalized patients during the study period

The monthly number of hospitalized patients during the three-year observation period is shown in Figure 2.

Boys accounted for 60% (n = 26) and girls for 40% (n = 17) of the study group, giving a male-to-female ratio of 1.5:1. The mean age was 7 years and 1 month (range: 23 months to 16 years and 1 month). Nonspecific symptoms suggestive of a preceding infection were reported in all children. These included fever in 93% (n = 40), cough in 70% (n = 30), nasal discharge in 58% (n = 25), diarrhea in 14% (n = 6), and vomiting in 9% (n = 4). The average interval between the onset of infection and muscle complaints was 4.5 days, with a maximum of 10 days. In 51% of cases, infectious symptoms were already subsiding at the time of admission. All patients experienced lower limb pain at admission or during hospitalization. One patient also reported upper limb pain; the rest had pain confined to the lower limbs. Gait disturbances occurred in 91% (n = 39) of patients, with 47% (n = 20) showing a transient complete refusal to bear weight. In 70% (n = 30), muscle symptoms appeared within 24 hours prior to admission, while the longest reported duration at presentation was four days. One patient developed typical BACM features only during hospitalization, having been admitted for severe infectious symptoms. Elevated CK activity was noted in 88% (n = 38) of patients. The mean initial CK level was 2,483 U/L (range: 54-24,077 U/L). Repeat measurements in some patients showed a mean peak CK of 2,843 U/L. In those retested before discharge, the mean CK decreased to 682 U/L. AST activity exceeded the reference range in 79% (n = 34), with a mean of 112 U/L (range: 22-527 U/L). Viral diagnostics were performed in selected cases, mainly with rapid antigen tests. An etiological agent was identified in 19 patients: 12 with influenza B, 5 with influenza A, 1 with both influenza A and B, and 1 with influenza A and RSV. No adenovirus or SARS-CoV-2 infections were detected. Only antigen tests were performed; PCR and serology were not used. Mean peak CK levels were 1,719 U/L in influenza A, 4,167 U/L in influenza B, and 1,749 U/L in cases without confirmed etiology. Leukopenia was observed in 40% (n = 18), neutropenia in 47% (n = 20), and thrombocytopenia in 16% (n = 7). Statistical analysis showed no significant effect of the identified pathogen on WBC or neutrophil counts. No patients developed dark urine. Dipstick urinalysis was positive for hemoglobinuria/myoglobinuria in four patients, but all showed erythrocytes on microscopy, and serum creatinine remained normal. In one case, the term “rhabdomyolysis” was initially used due to very high CK (24,077 U/L); however, retrospective review found insufficient grounds for this diagnosis. The same patient was hospitalized again a year later with BACM and referred to a metabolic disorders clinic for further evaluation. All patients received intravenous hydration. Analgesics (paracetamol, ibuprofen, or naproxen) were used in 65% (n = 28). Two patients required antibiotics for complications of the primary infection (pneumonia and severe otitis media). On average, lower limb pain and gait disturbances resolved within 2.6 days. In three patients, symptoms resolved within 24 hours; in one patient, they persisted for five days. Median hospital stay was three days. Key findings are summarized in Table 1.

**Table 1:** Summary of key findings

Feature	Value
Number of hospitalizations	43
Number of boys	25, including 1 hospitalized twice
Number of girls	16, including 1 hospitalized twice
Age	Average: 7 years 1 month (range: 23 months to 16 years 1 month)
Symptoms of preceding infection	43/43
Muscle pain	43/43
Gait disturbances	39/43
Elevated creatine kinase	38/43
Elevated aspartate aminotransferase	34/43
Creatine kinase on admission (normal: 0-180 U/L)	2,483 U/L (range: 54-24,077 U/L)
Leukopenia < 4,000 WBC	18/43
Neutropenia < 1,500 neutrophils	20/43
Urine discoloration/myoglobinuria	0/43
Length of hospitalization	Average: 3.4 days, median: 3 days
Time to symptom resolution	Average: 2.6 days

## Discussion

BACM has been described in the medical literature for nearly 70 years, most often in association with influenza virus infection [20]. While numerous detailed case reports exist, most retrospective studies involve only small to moderate patient cohorts collected over several years. Other studies included large cohorts [2,6,8,15,17,18,20,23,24], such as those by Brisca et al. [3] and Turan et al. [25], which included 113 and 114 patients, respectively.

The relative rarity of BACM contributes to limited clinical awareness among physicians. Although the condition usually presents with classic features and resolves spontaneously, it can still cause considerable anxiety for patients, families, and healthcare providers unfamiliar with its course [9,11]. On the one hand, international literature offers accessible descriptions of symptomatology and epidemiology; on the other, there are no standardized guidelines for clinical management. Consequently, physicians often rely on individual clinical experience, multidisciplinary knowledge, and recommendations proposed in the literature.

This study has several limitations. First, its retrospective design may have influenced data quality. Because no standardized management protocol was in place, particularly regarding diagnostic evaluation, not all patients underwent the same procedures. Only a portion of patients had swabs performed for pathogen identification by antigen or PCR testing. This reflects both limited availability and the gradual adoption of these methods, especially in the years following the COVID-19 pandemic. Second, some information relied on patient-reported data, which are inherently subjective. Finally, at the time of admission, patients may have been assessed by different members of a rotating medical team, which could also have affected consistency in data collection.

Following a review of studies conducted across different continents, our findings align closely with those reported by other research groups. Most children in our study were of preschool or early school age, consistent with the largest available studies, including Rosenberg et al. [24] (mean age 7.3 years), Turan et al. [25] (mean age 7 years), and Attaianese et al. [20] (mean age 6.6 years). The male predominance in our cohort (60%) falls within the reported range of 55%-86%, although most authors note a male-to-female ratio of 2:1 or higher. Consistent with prior reports, BACM showed a seasonal pattern, with over 50% of cases occurring in late autumn and winter [2,3,5,6,20]. Lower limb pain was present in all patients in our cohort, comparable to the reported range of 62%-100%, with at least five studies documenting myalgia in all cases. Gait disturbances occurred in 91% of our patients, similar to previously reported ranges of 46%-100%, with four studies noting this symptom in at least 87% of cases. Elevated serum CK was observed in most patients, with a mean of 2,483 U/L, consistent with literature values ranging from 943 to 4,066 U/L. Elevated AST occurred in most patients, with a mean of 112 U/L, corresponding to previously published mean values of 69-166 U/L. Leukopenia and neutropenia were observed in 40% and 47% of patients, respectively, comparable to reported ranges of 27%-76%. The average interval from onset of infection to appearance of myositis-related symptoms was 4.5 days in our cohort, similar to previously reported means of 2.4-7 days (median ~4.4 days). Muscle-related symptoms resolved on average within 2.6 days in our patients, which aligns with reported averages of 2.4-5 days; in at least two studies, resolution was vaguely reported as occurring within seven days.

Multiple literature reviews indicate that BACM is more frequently associated with influenza B infection. Our study supports this observation and further showed significantly higher CK levels in patients whose symptoms were triggered by influenza B [1,23]. Generalized post-infectious muscle pain is more typical in influenza B, although the clinical presentation of both influenza A and B is largely similar, with fever, rhinorrhea, and cough being the most common symptoms. Influenza B is more often linked to myositis, whereas influenza A is more frequently associated with neurological manifestations. However, clinical features alone are insufficient to reliably differentiate between the two virus types [7]. Öztürk et al. similarly found no difference in the incidence of leukopenia and neutropenia between influenza A and B infections [7]. In our cohort, the effect of oseltamivir on symptom resolution was not evaluated, as the drug was rarely administered. Most patients were admitted more than 48 hours after symptom onset, beyond the recommended therapeutic window for oseltamivir, limiting its potential effectiveness. Furthermore, given the self-limiting nature and excellent prognosis of BACM, routine antiviral therapy was not prioritized. Öztürk et al. also reported no significant difference in time to symptom resolution between patients treated with or without neuraminidase inhibitors. It remains possible, however, that symptoms resolve slightly faster in influenza-associated BACM compared to BACM triggered by other, often unidentified, viral pathogens [7]. Despite sometimes severe myalgia and alarming gait disturbances, all patients in our study achieved full recovery within one week, with management focused on bed rest, adequate hydration, and analgesia. Two patients experienced recurrent episodes, yielding a recurrence rate of 5%, which aligns with reported rates of 3%-10% [3]. Awareness of BACM improves patient care and helps avoid unnecessary diagnostic or therapeutic interventions. A reliable medical history, thorough physical examination including neurological assessment, and basic laboratory tests (CRP, CK, serum creatinine, urine dipstick) are generally sufficient to establish the diagnosis and exclude other serious conditions. Key differential diagnoses include Guillain-Barré syndrome, osteomyelitis, muscular dystrophy, and dermatomyositis. Rhabdomyolysis, in particular, should be considered a distinct and potentially life-threatening condition. Principal distinguishing features between these disorders and BACM are summarized in Table 2.

**Table 2:** Selected differential diagnoses for BACM Source: [13,16,21,25].

Condition	Distinguishing features	Diagnostic tools
Guillain-Barré syndrome	Lower limb weakness absent/decreased deep tendon reflexes, progressive symptoms onset 2-4 weeks post-infection	EMG, cerebrospinal fluid analysis
Osteomyelitis	Fever local swelling/redness history of skin trauma or procedures	Inflammatory markers, X-ray, ultrasound, MRI
Rhabdomyolysis	Severe general condition dark-colored urine history of trauma or exertion	CK, creatinine, potassium, phosphorus, calcium myoglobinuria
Muscular dystrophy	Family history chronic progressive weakness reduced deep tendon reflexes	Neurological exam, genetic testing
Dermatomyositis/polymyositis	Asymmetric involvement skin rash (e.g., Gottron papules), prolonged course joint involvement	Imaging (X-ray, US, MRI), inflammatory markers, autoimmune panel

The primary criteria for hospital admission in our cohort included complete loss of ambulation, severe myalgia, refusal of fluid intake, and other significant infectious manifestations. Individual cases were also assessed based on the extent of laboratory abnormalities at presentation. Parental anxiety, often strongly expressed and not always alleviated by the referring physician, also influenced admission decisions. According to several authors, when the clinical history and presentation are typical and initial evaluation excludes serious conditions, particularly AKI and rhabdomyolysis, hospitalization may not be strictly necessary, and outpatient management can be safe [3,13,20,23]. As attending physicians for some of the hospitalized patients, we observed no significant differences in disease course based on initial presentation, including gait disturbances or pain intensity. Symptom severity did not correlate consistently with serum CK levels. While elevated CK naturally raises concern for rhabdomyolysis and its potential complications, these remain exceedingly rare.

Following a detailed review, we concur that BACM is a self-limiting condition with an excellent prognosis and no long-term sequelae. Thorough communication with patients and caregivers is essential to alleviate unnecessary anxiety. In suspected BACM cases, we recommend initial laboratory evaluation, including complete blood count, CRP, CK, and urinalysis. These tests often show characteristic deviations that support the diagnosis and help exclude more serious conditions presenting with lower limb myalgia. Outpatient management appears feasible and safe for patients with partially preserved lower limb function and adequate response to over-the-counter analgesics. Such management should include rest, hydration, and symptomatic analgesia, supplemented with telephone and outpatient follow-up, including repeat laboratory testing. Children for whom history-taking and observation are unreliable due to age, those at high risk of falls, those with poor analgesic response or severe infectious symptoms, or cases with diagnostic uncertainty should be admitted for inpatient observation. Hospitalization should be limited to the period necessary to observe clinical improvement and to rule out serious conditions. Elevated CK reflects ongoing muscle inflammation and is a useful diagnostic indicator in suspected BACM, but it should not be the sole criterion for hospitalization. CK levels exceeding 5,000 U/L, particularly when accompanied by other laboratory abnormalities, warrant increased vigilance. Reassuringly, despite reports of CK levels as high as 54,000 U/L in other studies, no adverse clinical outcomes have been documented in the context of BACM.

## Conclusions

BACM is a rapidly resolving condition with an excellent prognosis that typically does not result in long-term consequences and, in most cases, does not require hospitalization. Given the characteristic symptoms, particularly the frequent refusal to walk, physicians should provide thorough explanations to patients and their families to reduce anxiety. For all suspected cases, we recommend assessment of biochemical parameters, including complete blood count, CRP, CK, and a general urine test, to support the initial diagnosis. The decision to hospitalize should be individualized, considering factors such as the patient’s age, general condition, pain severity, gait disturbances, presence of infectious symptoms, and results of additional tests.
